# Aquaporin 4 Suppresses Neural Hyperactivity and Synaptic Fatigue and Fine-Tunes Neurotransmission to Regulate Visual Function in the Mouse Retina

**DOI:** 10.1007/s12035-019-01661-2

**Published:** 2019-06-12

**Authors:** Yoko Ozawa, Eriko Toda, Hirohiko Kawashima, Kohei Homma, Hideto Osada, Norihiro Nagai, Yoichiro Abe, Masato Yasui, Kazuo Tsubota

**Affiliations:** 1 Laboratory of Retinal Cell Biology, Department of Ophthalmology, Keio University School of Medicine, 35 Shinanomachi, Shinjukuku, Tokyo, 160-8582 Japan; 2 Department of Ophthalmology, Keio University School of Medicine, 35 Shinanomachi, Shinjukuku, Tokyo, 160-8582 Japan; 3 Department of Pharmacology, Keio University School of Medicine, 35 Shinanomachi, Shinjukuku, Tokyo, 160-8582 Japan

**Keywords:** Aquaporin 4, Neural hyperactivity, Synaptic fatigue, Retina, Potassium, Glutamate

## Abstract

The bidirectional water channel aquaporin 4 (AQP4) is abundantly expressed in the neural tissue. The advantages and disadvantages of AQP4 neural tissue deficiency under pathological conditions, such as inflammation, and relationship with neural diseases, such as Alzheimer’s disease, have been previously reported. However, the physiological functions of AQP4 are not fully understood. Here, we evaluated the role of AQP4 in the mouse retina using *Aqp4* knockout (KO) mice. Aqp4 was expressed in Müller glial cells surrounding the synaptic area between photoreceptors and bipolar cells. Both scotopic and photopic electroretinograms showed hyperactive visual responses in KO mice, gradually progressing with age. Moreover, the amplitude reduction after frequent stimuli and synaptic fatigue was more severe in KO mice. Glutamine synthetase, glutamate aspartate transporter, synaptophysin, and the inward potassium channel *Kir2.1*, but not *Kir4.1*, were downregulated in KO retinas. KIR2.1 colocalized with AQP4 in Müller glial cells at the synaptic area, and its expression was affected by *Aqp4* levels in primary Müller glial cell cultures. Intraocular injection of potassium in wild-type mice led to visual function hyperactivity, as observed in *Aqp4* KO mice. Mitochondria molecules, such as *Pgc1α* and *CoxIV*, were downregulated, while apoptotic markers were upregulated in KO retinas. AQP4 may fine-tune synaptic activity, most likely by regulating potassium metabolism, at least in part, via collaborating with KIR2.1, and possibly indirectly regulating glutamate kinetics, to inhibit neural hyperactivity and synaptic fatigue which finally affect mitochondria and cause neurodegeneration.

**Electronic supplementary material:**

The online version of this article (10.1007/s12035-019-01661-2) contains supplementary material, which is available to authorized users.

## Introduction

The bidirectional water channel aquaporin 4 (AQP4) is widely distributed in the plasma membrane and maintains the tissue microenvironment by supporting molecular transfer between extracellular and intracellular spaces [1–3]. Although the vulnerability or advantages of its deficiency in neural tissue have been reported under pathological conditions [4–8], its physiological function is not fully understood [9].

The aquaporin family (AQP0–12) has been well investigated in the kidney [1, 2, 10, 11], where water permeability is well controlled in each part of the nephron; each AQP is localized in a specific part of the nephron to regulate water kinetics. Among AQPs, AQP4 is a polypeptide encoded by a brain-derived cDNA and constitutes the predominant water channel protein in neural tissue [10]. AQP4 regulates extracellular space volume, waste clearance, and calcium ion signaling, as well as potassium buffering [12]. Its expression is reportedly downregulated in pathological lesions in neuromyelitis optica, an inflammatory and necrotizing neural disease clinically characterized by selective involvement of the optic nerves and spinal cord [13]. Serum anti-AQP4 autoantibodies are frequently found in severe, recurrent autoimmune optic neuritis, namely neuromyelitis optica spectrum disorder [14], and may determine the disease severity [15]. AQP4 protein is increased in the intraocular fluid of diabetic patients [16], and its relationship with diabetes is well reported in mice [6, 7, 10, 17]. AQP4 is induced in the retina of diabetic mice [18] and suppresses inflammation [17]. Another report showed that, while AQP4 is increased in the retina of these mice, the potassium channel subunit KIR4.1 is downregulated, thus inducing disruption of the blood-retina barrier and resulting in edema and inflammation [19]. Colocalization of AQP4 and KIR4.1 in astrocyte end feet has been shown to regulate blood-brain barrier function, and the relationship between AQP4 and blood vessels is well accepted [20, 21].

In contrast, while AQP4 is expressed in neural tissue, its impact on neural functions is still under debate. AQP4 may mediate the clearance of amyloid beta peptides related to Alzheimer’s disease [22] and regulate extracellular space volume during synaptic activity [23]. In the retina, AQP4 is expressed in Müller glial cells [24] which not only provide structural support but also maintain homeostasis of the retinal microenvironment and regulate neuronal activity [25]. A retinal phenotype of *Aqp4* deficiency in mice was reported using electroretinogram (ERG), which measures visual function; the function was comparable between wild-type (WT) and knockout (KO) mice at early time point after birth, postnatal day 17 [8], but a clear impairment was observed in aged mice at 10 months [26]. However, the development of this neural dysfunction and the underlying mechanisms remain obscure.

In the current study, we explored the impact of AQP4 in keeping neuronal homeostasis and synaptic function in the mouse retina. For this purpose, we assessed AQP4 expression in the retina, performed ERG in relatively young adult *Aqp4* KO mice to evaluate visual function, and examined the expression of synaptic markers, mitochondria molecules, and apoptotic markers.

## Materials and Methods

### Animals

Male *Aqp4* KO mice generated as described previously [5, 27] (acc. no. CDB0758 K: http://www.cdb.riken.jp/arg/mutant%20mice%20list.html), and back crossed to C57B6J background, were maintained in an air-conditioned room (22 °C) under a 12-h light/dark cycle (lights on from 8 a.m. to 8 p.m.) with free access to food and water, at the animal facility of Keio University School of Medicine. All animal experiments were conducted in accordance with the ARVO Statement for the Use of Animals in Ophthalmic and Vision Research and the guidelines of the Animal Care Committee of Keio University. For each experiment, KO and WT mice were serially numbered, and the examiners were blind to genotype when recording the results at the time of experiments. Overall, no clear differences in body weight and size were observed in KO mice (data not shown).

### Immunohistochemistry

Mouse eyes were enucleated and fixed in 4% paraformaldehyde overnight at 4 °C. After fixation, the eyes were embedded in paraffin (Sakura Finetek Japan, Tokyo, Japan), and 6- to 8-μm-thick sections including the optic nerve head to the most peripheral region of the retina were prepared and deparaffinized. The sections were blocked with TNB blocking buffer (0.1 M Tris-HCl [pH 7.5] and 0.15 M NaCl) for 30 min at room temperature and then incubated overnight with antibodies for AQP4 (Sigma-Aldrich, St. Louis, MO; or the one generated by Ramadhanti et al.) [28], glutamine synthetase (GS; Millipore, Burlington, MA), synaptophysin (DAKO, Agilent Technologies, Santa Clara, CA), postsynaptic density protein 95 (PSD95; Thermo Fisher Scientific, Waltham, MA), bassoon (Enzo Biochem, Inc., Farmingdale, NY), and KIR 2.1 (Abcam, Cambridge, UK). Then, sections were incubated with Alexa 488-conjugated or Alexa 555-conjugated secondary antibodies (Invitrogen Japan, Tokyo, Japan) with subsequent counterstaining with DAPI solution (2 μg/mL) for 1 h at room temperature. Sections from eight animals were examined under a microscope equipped with a digital camera (Olympus Co., Tokyo, Japan), and fluorescent images were obtained using a confocal microscope (TCS-SP5; Leica, Tokyo, Japan).

### ERG Recordings

Mice were dark-adapted for at least 12 h and then placed under dim-red illumination before conducting the ERGs. Mice were anesthetized with intraperitoneal combined anesthetics [midazolam 4 mg/kg of body weight (BW) (Sandoz Japan, Tokyo, Japan), medetomidine 0.75 mg/kg BW (Nippon Zenyaku Kogyo Co., Ltd., Fukushima, Japan), butorphanol tartrate 5 mg/kg BW (Meiji Seika Pharma Co., Ltd., Tokyo, Japan)] and kept on a heating pad throughout the experiment. Mouse pupils were dilated using a single drop of a mixture of tropicamide and phenylephrine (0.5% each; Mydrin-P®; Santen, Osaka, Japan). The ground and reference electrodes were then placed on the tail and in the mouth, respectively, while the active gold wire electrodes were placed on the cornea.

Recordings were made using a PowerLab System 2/25 (AD Instruments, New South Wales, Australia). Full-field scotopic ERGs were measured in response to a flash stimulus at intensities ranging from − 2.1 to 2.9 log cd s/m^2^. Photopic ERGs were measured after 10 min of light adaptation. Flash stimuli ranging from 0.4 to 1.4 log cd s/m^2^ were used for recordings with a background of 30 cd s/m^2^ (PowerLab System 2/25, AD Instruments, New South Wales, Australia), and the results of 20 single-flash trace trials were averaged. Dark-adapted flicker ERGs [29] were recorded using repeated stimuli at 0.4 log cd s/m^2^ with a frequency ranging from 0.5 to 30 Hz for 600 ms, and the first and 20th waves of continuous recordings were analyzed. The responses were differentially amplified and filtered through a digital bandpass filter ranging from 0.3 to 1000 Hz. Each stimulus was delivered using a commercial stimulator (Ganzfeld System SG-2002; LKC Technologies, Inc., Gaithersburg, MD). The a-wave amplitude was measured from the baseline to the trough, while the b-wave amplitude was measured from the trough of the a-wave to the peak of the b-wave. The implicit times of the a- and b-waves were measured from the onset of the stimulus to the peak of each wave. The peak points were automatically indicated by the system and confirmed by the examiner. In each recording, four to five KO and WT animals were used.

### Real-Time Reverse Transcription-Polymerase Chain Reaction

Total RNA was isolated from mouse retinas at 16 weeks of age using TRIzol reagent (Life Technologies, Carlsbad, CA, USA). RNA concentration was measured using NanoDrop 1000 (Thermo Fisher Scientific), and 1 μg RNA was reverse-transcribed using the SuperScript VILO master mix (Life Technologies, Carlsbad, CA, USA), according to the manufacturer’s instructions. The following primer sequences were used: glyceraldehyde 3-phosphate dehydrogenase (*Gapdh*), forward 5′-AACTTCGGCCCCATCTTCA-3′ and reverse 5′-GATGACCCTTTTGGCTCCAC-3′; *GS*, forward 5′-ACTGTGAGCCCAAGTGTGTG-3′ and reverse 5′-GGAGGTACATGTCGCTGTTG-3′; glutamate aspartate transporter (*Glast*), forward 5′-GAGCCTCACCAAGGAAGATG-3′ and reverse 5′-CCTCCCGGTAGCTCATTTTA-3′; synaptophysin, forward 5′-GCATTGCTGATGCTGCTG-3′ and reverse 5′-CACCTTCACGAAGCCAAGG-3′; peroxisome proliferator-activated receptor gamma coactivator 1-alpha (*Pgc1α*), forward 5′-GATGAATACCGCAAAGAGCA-3′ and reverse 5′-AGATTTACGGTGCATTCCT-3′; cytochrome c oxidase subunit 4 (*CoxIV*), forward 5′-CGACTGGAGCAGCCTTTCC-3′ and reverse 5′-CTGTTCATCTCGGCGAAGC-3′; cytochrome c (*CytC*), forward 5′-CCAGGCTGCTGGATTCTCTTACACA-3′ and reverse 5′-TCCAAATACTCCATCAGGGTATCC-3′; heme oxygenase 1 (*Ho-1*), forward 5′-ACGCATATACCCGCTACCTG-3′ and reverse 5′-CCAGAGTGTTCATTCGAGCA-3′; fission 1 (*Fis1*), forward 5′-ATATGCCTGGTGCCTGGTTC-3′ and reverse 5′-AGTCCCGCTGTTCCTCTTTG-3′; mitofusin 1 (*Mfn1*) forward 5′-GATGTCACCACAGAGCTGGA-3′ and reverse 5′-AGAGCCGCTCATTCACCTTA-3′; mitofusin 2 (*Mfn2*), forward 5′-CCCCTCTCAAGCACTTTGTC-3′ and reverse 5′-ACCTGCTCTTCCGTGGTAAC-3′; sulfonylurea receptor 1 (*Sur1*) forward 5′-ctgctctttgtcctggtgtg-3′ and reverse 5′-cagctggcatgtacaaatgg; and potassium channel, subfamily K, member 3 (also known as *Task-1*), forward 5′-gctccttctacttcgccatc-3′ and reverse 5′-gaacatgcagaacaccttgc. Taqman probes (Applied Biosystems, Thermo Fisher Scientific) were used for *Kir2.1* (also known as *Kcnj2*, potassium inwardly rectifying channel, subfamily J, member 2; Mm00434616), *Aqp4* (Mm00802131), *Kir4.1* (also known as *Kcnj10*, potassium inwardly rectifying channel, subfamily J, member 10; Mm00445028), potassium channel, subfamily V, member 2 (*Kcnv2*; Mm00807577), b cell lymphoma 2 (bcl2; Mm00477631), and BCL2-associated X protein (*Bax*; Mm00432050). Real-time PCR was performed using the StepOnePlus™ PCR system (Applied Biosystems, Thermo Fisher Scientific), and gene expression was quantified using the ΔΔCT method. All mRNA levels were normalized to those of *Gapdh*. For expression analysis, five to ten KO and WT animals or four to five culture samples with or without knockdown (KD) of *Aqp4* (see below) were analyzed. All the real-time reverse transcription-polymerase chain reaction (RT-PCR) reactions using the SYBR systems were performed after validating the primers by checking whether the melting curves show a single peak (data not shown), and those using Taqman probes were done following the manufacturer’s protocol.

### Cell Culture

Primary Müller glial cell culture was performed as previously reported [30] with some modifications. Briefly, Müller glial cells were isolated from the retinas of C57BL/6 mice at postnatal days 7–12. After the eyes were enucleated, the retinas were isolated and incubated in 0.1% trypsin/EDTA (Gibco, Thermo Fisher Scientific) at 37 °C for 30 min. Retinal cells were dissociated by pipetting and maintained for a few days in Dulbecco’s modified Eagle’s medium containing high glucose (4500 mg/L) and Ham’s nutrient mixture F-12 medium (Gibco, Carlsbad, CA) supplemented with 10% fetal bovine serum, penicillin (100 units/mL), and streptomycin (100 μg/mL), at 37 °C, under a humidified atmosphere with 5% CO_2_, to selectively purify Müller glial cells.

To knockdown *Aqp4* in Müller glial cells, we introduced *Aqp4* siRNA (MSS202139, Thermo Fisher Scientific), while control siRNA (Negative Control Lo GC, Thermo Fisher Scientific) was introduced as negative control, using Lipofectamine RNAiMAX Reagent (Thermo Fisher Scientific), according to the manufacturer’s protocol. Cells were incubated for 24 h before collecting them for experiments. The cells were then washed with 1 mL PBS, sonicated in an ice bath, and placed in TRIzol reagent (Life Technologies) for mRNA extraction and real-time RT-PCR analyses (see above).

### Intravitreal Injection of KCL

Six-week-old C57BL/6 male mice (CLEA Japan, Tokyo, Japan) were anesthetized with intraperitoneal injection of combined anesthetics {midazolam 4 mg/kg BW (Sandoz Japan, Tokyo, Japan), medetomidine 0.75 mg/kg BW (Nippon Zenyaku Kogyo Co., Ltd., Fukushima, Japan), butorphanol tartrate 5 mg/kg BW (Meiji Seika Pharma Co., Ltd., Tokyo, Japan)]; their pupils were dilated using a single drop of a mixture of tropicamide and phenylephrine (0.5% each; Mydrin-P®; Santen Pharmaceutical Co., Ltd., Osaka Japan) and received 2-μL intravitreal injections of either KCL dissolved in PBS (50 mM) or just PBS as vehicle via an UltraMicroPump (type UMP2) equipped with a MicroSyringe Pump Controller (World Precision Instruments, Sarasota, FL) [31]. Twelve animals were used in each group.

### Statistical Analysis

All results are expressed as the mean ± standard deviation. Student’s *t* test was used to assess the statistical significance of differences among the groups of animals or cell culture conditions, with *p* < 0.05 regarded as significant.

## Results

### AQP4 Localization at the Synaptic Area of the Retina

**Fig. 1:**
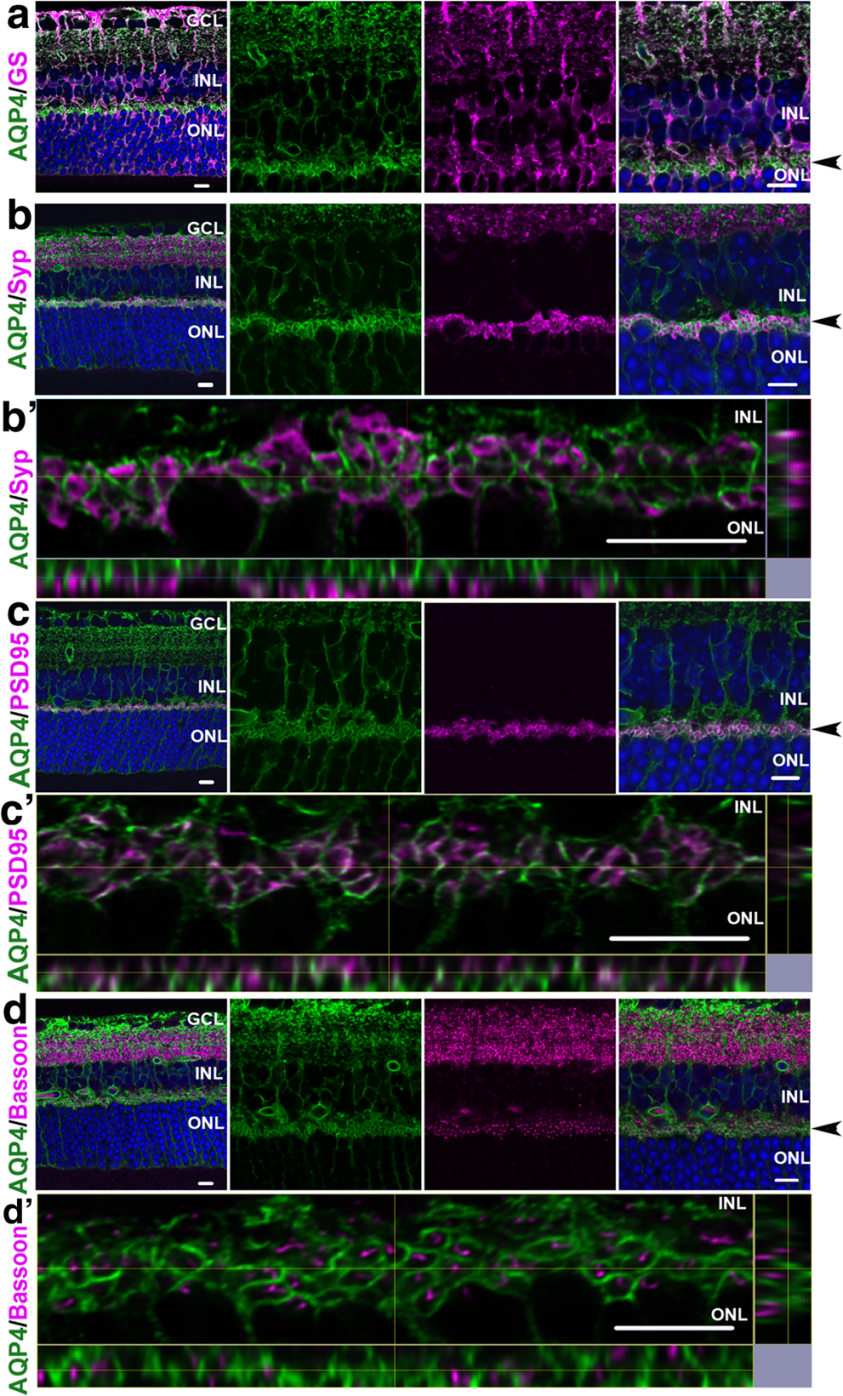
AQP4 localization at the synaptic area of the mouse retina. a–d Wild-type retinal sections immunostained with AQP4 and glutamine synthetase (GS; a), synaptophysin (Syp; b, b′), PSD95 (c, c′), or bassoon (d, d′). Arrowheads show the outer plexiform layer (OPL) where photoreceptor-bipolar synapses are distributed. b′, c′, and d′ are magnifications of b, c, and d, respectively. AQP4 is coexpressed with GS but not with Syp, PSD95, or bassoon. Bassoon signals are surrounded by AQP4 (d, d′). *n* = 8. GCL ganglion cell layer, INL inner nuclear layer, ONL outer nuclear layer. Scale bars, 10 μm

AQP4 was coexpressed with GS, a Müller glial cell marker, in the retina of WT mice (Fig. 1a). Müller glial cells span the entire thickness of the neural retina [25], and AQP4 was abundantly observed in the inner layer. In addition, AQP4 expression was also observed in the outer plexiform layer (OPL) where photoreceptor-bipolar synapses are distributed. Double immunostaining with the synaptic markers’ synaptophysin (Fig. 1b, b′), PSD95 (Fig. 1c, c′), and bassoon (Fig. 1d, d′) showed no colocalization with AQP4. In particular, bassoon signals were surrounded by AQP4 signal (Fig. 1d, d′), suggesting that AQP4 is localized in Müller glial cells surrounding the synapses between photoreceptor and bipolar cells.

### Visual Function Hyperactivity in *Aqp4* KO Mice

**Fig. 2:**
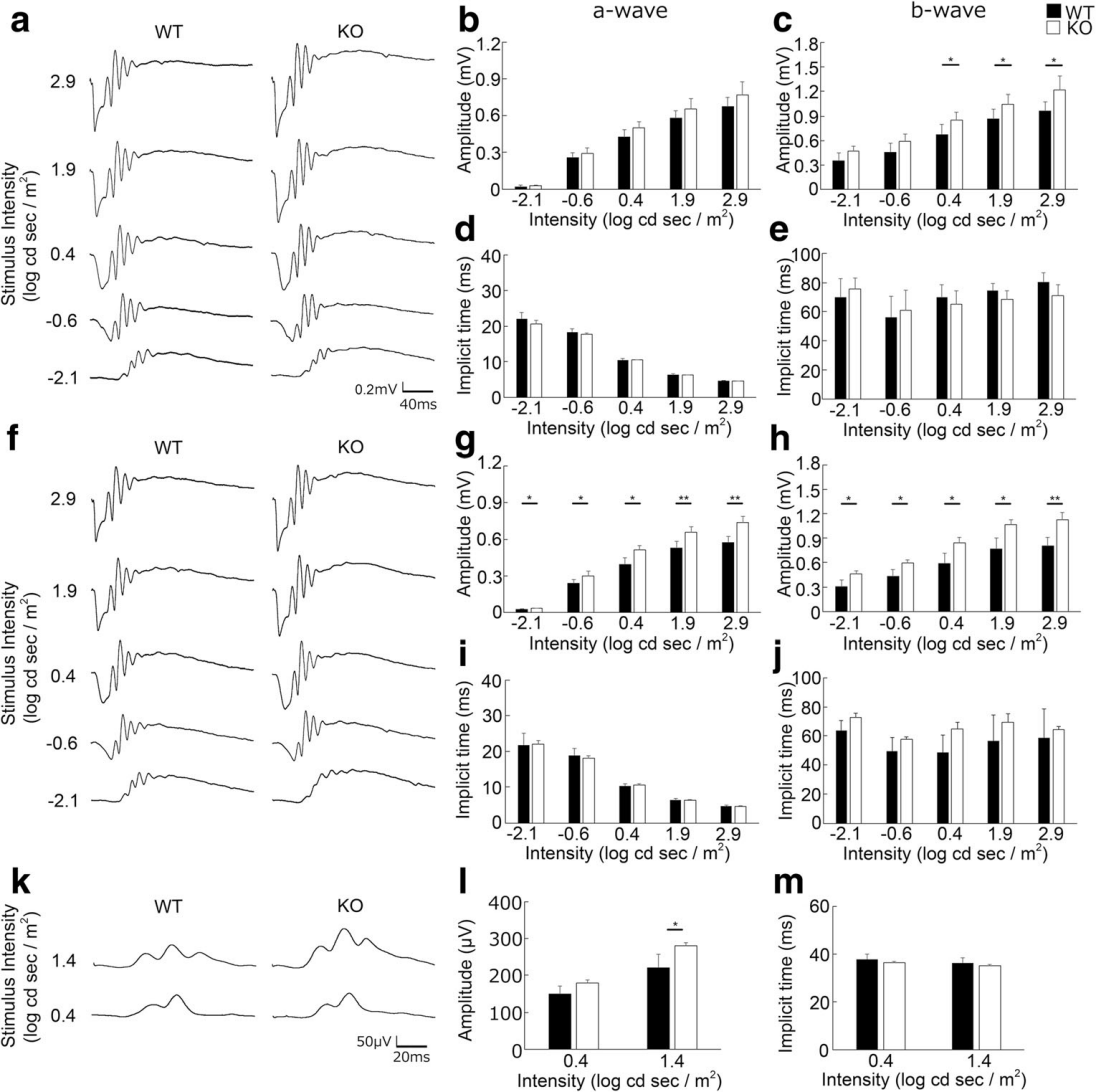
Visual function hyperactivity in *Aqp4* knockout (KO) mice. Electroretinogram responses in wild-type (WT) and *Aqp4* KO mice at 12 (**a**–**e**) and 16 (**f**–**m**) weeks of age. Scotopic (**a**–**j**) and photopic (**k**–**m**) responses. Representative wave forms from an individual mouse (**a**, **f**, **k**) and mean data of a-wave (**b**, **d**, **g**, **i**) and b-wave (**c**, **e**, **h**, **j**, **l**, **m**) are shown. The scotopic b-wave amplitude is higher in *Aqp4* KO mice than in WT mice both at 12 (**c**) and 16 (**h**) weeks of age, while the scotopic a-wave (**g**) and photopic b-wave (**l**) amplitudes are higher in *Aqp4* KO than WT mice at 16 weeks of age. There are no changes in the implicit time (**d**, **e**, **i**, **j**, **m**). Twelve-week-old mice, *n* = 5 (WT) and 5 (KO); 16-week-old mice, *n* = 4 (WT) and 5 (KO). *p < 0.05

The visual function of *Aqp4* KO mice was recorded by scotopic and photopic ERGs. In general, the a-wave represents photoreceptor function, while the b-wave shows the postsynaptic inner retinal function transferred from photoreceptor activity. We found that b-wave amplitude was higher in 12-week-old KO mice than in WT mice of the same age (WT and KO; *n* = 5, 5; *p* < 0.05; Fig. 2a–e, Supplementary Table 1), and moreover, a-wave and b-wave amplitudes were also higher in KO than in WT mice at 16 weeks of age (*n* = 4, 5; *p* < 0.05 and *p* < 0.01, respectively; Fig. 2f–j, Supplementary Table 1). No change was observed in 8-week-old mice (data not shown). Photopic ERG also showed an increase in b-wave amplitude in 16-week-old KO mice compared with that in WT mice (*n* = 4, 5; *p* < 0.05; Fig. 2k–m, Supplementary Table 1). There were no changes in the implicit time of each recording. Meanwhile, there were no obvious histological changes including retinal thickness in *Aqp4* KO mice (data not shown).

### Decrement of Visual Responses After Repeated Stimuli in *Aqp4* KO Mice

**Fig. 3:**
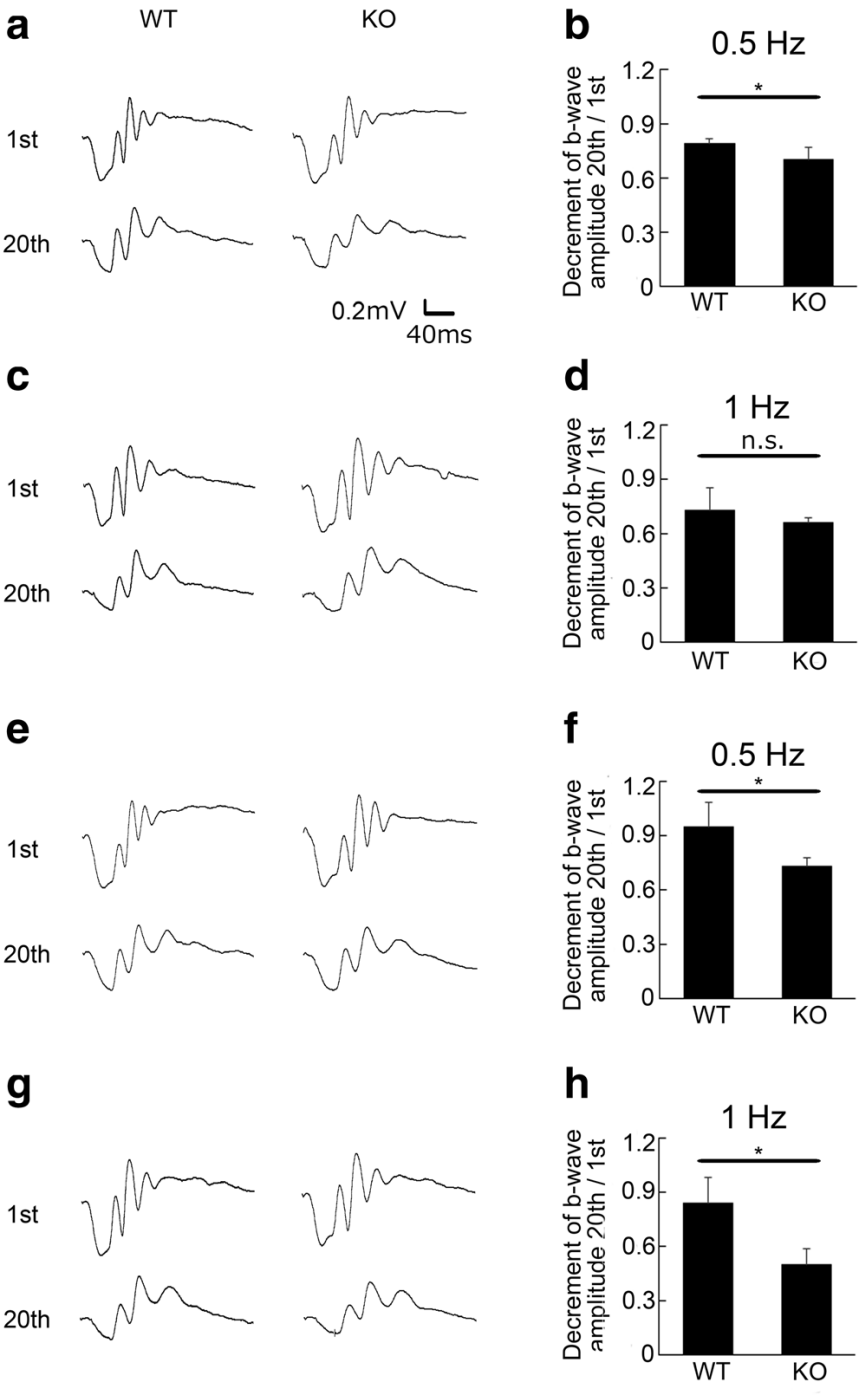
Decrement of visual responses after repeated stimuli in *Aqp4* knockout (KO) mice. Scotopic electroretinogram responses using flicker stimuli from wild-type (WT) and *Aqp4* KO mice at 10 (**a**–**d**) and 12 (**e**–**h**) weeks of age. Mice were stimulated with 0.5-Hz (**a**, **b**, **e**, **f**) or 1-Hz (**c**, **d**, **g**, **h**) flicker light. Representative wave forms from an individual mouse (**a**, **c**, **e**, **g**) and the mean b-wave amplitude ratio of the 20th to the 1st stimulus (**b**, **d**, **g**, **i**) are shown. The ratio is smaller in *Aqp4* KO than WT mice at 10 weeks of age with 0.5-Hz stimuli (**a**, **b**) and at 12 weeks with 0.5-Hz (**e**, **f**) and 1-Hz stimuli (**g**, **h**). Ten-week-old mice, *n* = 5 (WT) and 6 (KO); 12-week-old mice, *n* = 4 (WT) and 6 (KO). *p < 0.05

To elucidate whether *Aqp4* deficiency could affect the recovery of synaptic homeostasis in retinal neurons of *Aqp4* KO mice, ERG responses were recorded using flicker stimuli (Fig. 3a–h). The ratio of the scotopic ERG b-wave amplitude at the 20th stimulus to that of the first stimulus of 0.5 Hz was smaller in KO than in WT mice both at 10 weeks (WT 0.80 ± 0.03, *n* = 5, and KO 0.71 ± 0.07, *n* = 6; *p* = 0.023; Fig. 3a, b) and at 12 weeks of age (WT 0.95 ± 0.14, *n* = 4; KO 0.73 ± 0.05, *n* = 6; *p* = 0.042; Fig. 3e, f). The latter was also true for 1-Hz stimuli (WT 0.84 ± 0.15, *n* = 4; KO 0.50 ± 0.09, *n* = 6; *p* = 0.013; Fig. 3g, h), indicating a greater decrement in *Aqp4* KO mice after repeated stimuli. These data suggested that the electrical potential of neurons may not return to the appropriate resting potential after firing due to the insufficient clearance of synaptic mediators in *Aqp4* KO mice.

### Alterations in mRNA Levels Related to Synaptic Transmission in the Retina of *Aqp4* KO Mice

**Fig. 4:**
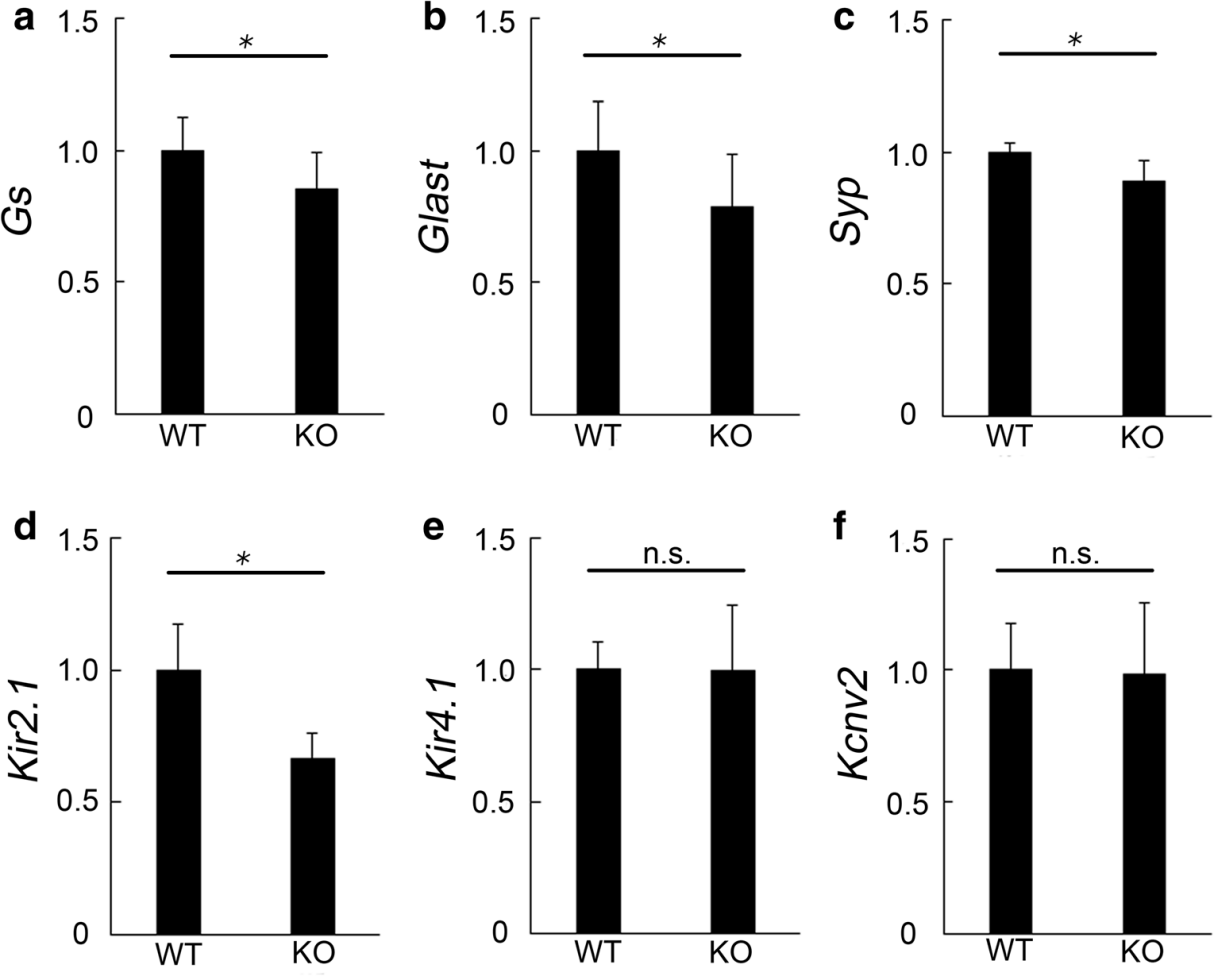
Alteration in mRNA levels related to synaptic transmission in the retina of *Aqp4* knockout (KO) mice. Real-time RT-PCR in retinal samples derived from wild-type (WT) and *Aqp4* KO mice at 16 weeks of age (**a**–**f**). The relative mRNA levels of glutamine synthetase (*Gs*; **a**), glutamate aspartate transporter (*Glast*; **b**), synaptophysin (*Syp*; **c**), and *Kir2.1* (**d**) are downregulated in the retina of *Aqp4* KO mice, compared with the respective levels in WT retinas. The levels of *Kir4.1* (**e**) and *Kcnv2* (**f**) are not changed. *n* = 5–10. *p < 0.05

The levels of functional molecules related to synaptic transmission were measured by real-time PCR in the retina of *Aqp4* KO mice. Significantly lower levels of *Gs* (WT and KO, *n* = 7, 9; *p* = 0.046; Fig. 4a) and *Glast* (*n* = 9, 10; *p* = 0.029; Fig. 4b), which are expressed in Müller glial cells and contribute to neurotransmitter metabolism and transfer, respectively, were found in the retina of *Aqp4* KO than of WT mice at 16 weeks of age. The presynaptic molecule *synaptophysin* was also downregulated in KO mice (*n* = 5, 5; *p* = 0.036; Fig. 4c).

At the synaptic area, potassium channels are gated by intracellular calcium elevation and are associated with synaptic plasticity [32]. The extracellular potassium released by excitatory neurons is buffered by glial cells in the brain [33]. Real-time PCR analysis showed that the mRNA levels of one of the strongly rectifying inward potassium channels, *Kir2.1* [32, 34], was downregulated in the neural retinal tissue of *Aqp4* KO mice (*n* = 9, 9; *p* = 0.000; Fig. 4d). In contrast, the mRNA expression of *Kir4.1*, a weakly rectifying channel acting both as an inward and outward channel [32, 34] (*n* = 5, 5; *p* = 0.990; Fig. 4e); *Kcnv2*, a voltage-gated potassium channel subunit expressed in photoreceptor cells [35] (*n* = 5, 5; *p* = 0.917; Fig. 4f); and *Sur1* (*n* = 5, 5; *p* = 0.379, data not shown) and *TASK-1* (*n* = 5, 5; *p* = 0.828, data not shown), two potassium channels both of which are found in the retina [36], was not changed by *Aqp4* deletion.

### *Kir2.1 *Downregulation in Müller Glial Cells upon *Aqp4* Deletion

**Fig. 5:**
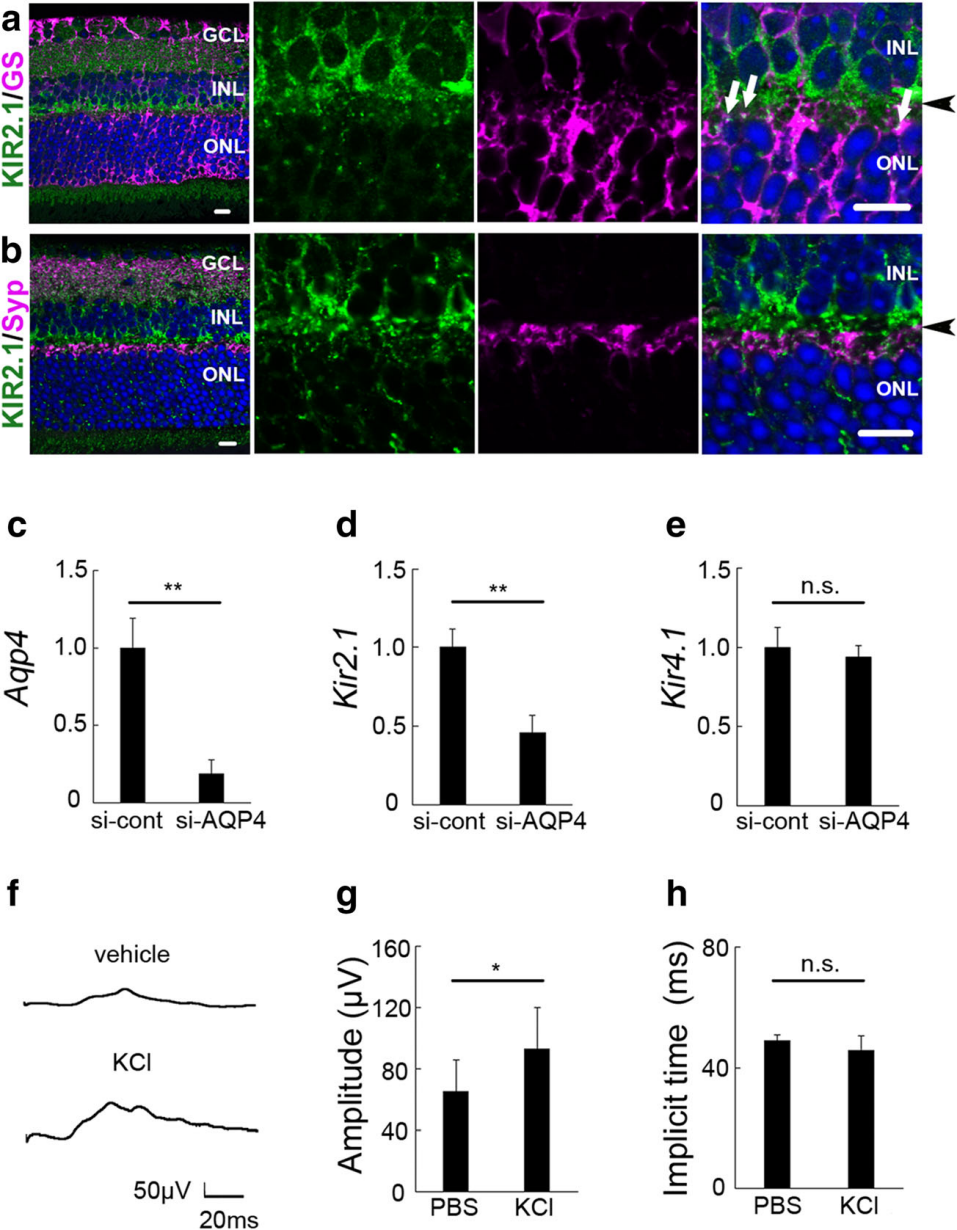
Modulation of intraocular potassium levels induces hyperactive photopic electroretinogram (ERG) responses in mice. a, b Double immunohistostaining for KIR2.1 and glutamine synthetase (GS; a, a′) or synaptophysin (Syp; b, b′) in wild-type (WT) retinal sections. a′ and b′ are magnifications of a and b, respectively. GS (a, a′; arrows) but not Syp (b, b′) colocalizes with KIR2.1. Arrowheads show the outer plexiform layer. *n* = 8. GCL ganglion cell layer, INL inner nuclear layer, ONL outer nuclear layer. Scale bar, 10 μm. c–e Real-time RT-PCR of primary Müller glial cell culture samples transfected with control (si-cont) or *Aqp4* siRNA (si-AQP4). Quantification of the relative mRNA levels confirms the knockdown (KD) of *Aqp4* (c). *Kir2.1* (d) but not *Kir4.1* (e) is downregulated in Müller glial cells. *n* = 4 (control) and 5 (KD). f–h Photopic ERGs after intraocular injection of vehicle (PBS) or KCL. The b-wave amplitude is increased by the potassium load (f, g). The implicit time is not changed (H). *n* = 12 for each group; *p < 0.05; **p < 0.01

Double immunohistochemistry showed that KIR2.1 localized in GS-positive Müller glial cells (Fig. 5a, a′). Meanwhile, KIR2.1 did not colocalize with synaptophysin expressed by neuronal cells in the OPL (Fig. 5b, b′). To examine whether *Kir2.1* expression is affected by *Aqp4* deletion in Müller glial cells, we used a primary culture system. In cultured Müller glial cells, *Aqp4* knockdown (KD), confirmed by real-time PCR (control and KD, *n* = 4, 5; *p* = 0.002; Fig. 5c), resulted in downregulation of *Kir2.1* mRNA levels (*n* = 4, 5; *p* = 0.000; Fig. 5d), but not of *Kir4.1* (*n* = 4, 5; *p* = 0.444; Fig. 5e), suggesting that *Kir2.1* and not *Kir4.1* levels are affected by *Aqp4* deletion in Müller glial cells.

### Modulation of Intraocular Potassium Levels Induces Hyperactive Visual Function in *Aqp4* KO Mice

Since KIR2.1 acts as an inward potassium channel, its downregulation and the subsequent increase in extracellular potassium levels may be associated with the hyperactivity of visual responses observed in KO mice. To further examine this, we intraocularly injected KCl and measured photopic ERG; for technical reasons, we could not measure scotopic ERG, as it requires dark adaptation, and the injection needed to be done under bright light. The amplitude of photopic ERG stimulated by 1.4 log cd s/m^2^ increased after potassium loading, in a similar manner as after *Aqp4* deletion in mice (vehicle 65.24 ± 20.33 mV; KCL 92.81 ± 26.99 mV; *n* = 12, *p* = 0.010; Fig. 5f, g). The implicit time was not changed (Fig. 5f, h).

### Mitochondria Changes in the Retina of *Aqp4* KO Mice

**Fig. 6:**
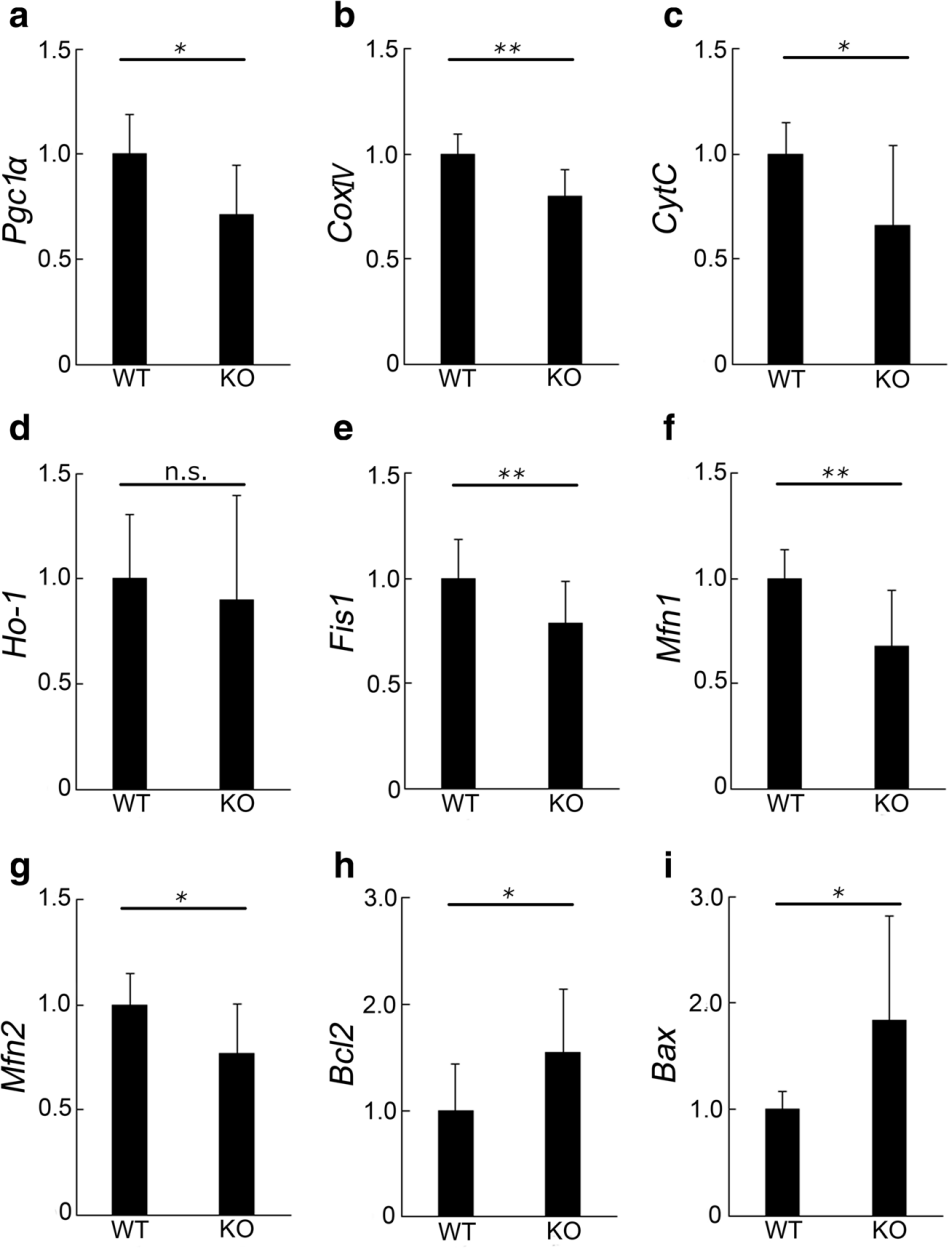
Mitochondria changes in the retina of *Aqp4* knockout (KO) mice. Real-time RT-PCR in retinal samples derived from wild-type (WT) and *Aqp4* KO mice at 16 weeks of age (**a**–**i**). The relative mRNA levels of *Pgc1α* (**a**), *CoxIV* (**b**), *CytC* (**c**), *Fis1* (**e**), *Mfn1* (**f**), and *Mfn2* (**g**) are downregulated in *Aqp4* KO retinas, while *Ho-1* (**d**) levels are not changed, compared with the respective levels in WT mice. The mRNA levels of *Bcl2* (**h**) and *Bax* (**i**) are higher in *Aqp4* KO than in WT retina. *n* = 5–10. *p < 0.05; **p < 0.01

In contrast to the increased ERG responses, the expression of functional molecules in Müller glial cells and neurons was decreased, as shown above. We next analyzed the mRNA levels of mitochondria-related molecules. Mitochondria constitute the energy supplier of the cells, and the retina is an energy-demanding tissue due to high neuronal activity [37, 38]. In the retina of *Aqp4* KO mice at 16 weeks of age, we found a significant downregulation of the mRNA levels of *Pgc1a*, an indispensable molecule for mitochondria biogenesis (WT and KO, *n* = 7, 9; *p* = 0.018; Fig. 6a), and *CoxIV* (*n* = 5, 5; *p* = 0.022; Fig. 6b) and *CytC* (*n* = 8, 10; *p* = 0.024; Fig. 6c), two mitochondrial respiratory enzymes, compared with the respective levels in the retina of WT mice. Meanwhile, the levels of *Ho-1*, an antioxidative molecule induced by oxidative stress and related to respiratory responses in mitochondria to resolve the stress, were not changed (*n* = 8, 10; *p* = 0.610; Fig. 6d), suggesting no increase in oxidative stress. In addition, the levels of *Fis1* (*n* = 9, 10; *p* = 0.002; Fig. 6e), *Mfn1* (*n* = 7, 9; *p* = 0.008; Fig. 6f), and *Mfn2* (*n* = 9, 10; *p* = 0.020; Fig. 6g), which are induced during mitochondria remodeling to eliminate pathological mitochondria, were reduced. In contrast, the apoptosis checkpoint family molecules, *Bcl2* (*n* = 8, 10; *p* = 0.042; Fig. 6h) and *Bax* (*n* = 8, 10; *p* = 0.025; Fig. 6i) were upregulated, suggesting that mitochondria homeostasis is disorganized in the retina of *Aqp4* KO mice.

## Discussion

In this study, we demonstrated that AQP4 is expressed in Müller glial cells at the synaptic area between photoreceptors and bipolar cells, which compose the OPL. Compared with WT mice, *Aqp4* KO mice displayed hyperactive visual function, which gradually progressed with age. The decrement in b-wave amplitudes after repeated stimuli was also greater in *Aqp4* KO mice. Functional molecules related to synaptic transmission were downregulated in KO retinas. In addition, KIR2.1, an inward potassium channel, expressed at the synaptic area in Müller glial cells, was also downregulated, while increased extracellular potassium levels in WT mice recapitulated the hyperactive photopic ERG responses in KO animals. In *Aqp4* KO retinas, molecules related to mitochondria biogenesis, respiration, and remodeling were downregulated, whereas apoptotic markers were upregulated.

The presence of AQP4 in Müller glial cells shown by immunostaining is consistent with a previous report using immune electron microscopy images [24]. This study found that AQP4 is concentrated at the perivascular end feet of Müller glial cells. Additionally, we observed that AQP4 is also distributed around, but not colocalized with, neuronal synapses between photoreceptor and bipolar cells.

*Aqp4* KO mice did not show obvious histological abnormalities at the analyzed ages (data not shown), consistently with previous reports [9]; however, they exhibited a clear phenotype in visual responses recorded by ERG. KO mice showed hyperactivity both in scotopic and photopic ERGs, which reflect rod and cone photoreceptor pathways functioning under dark and light conditions, respectively. Moreover, the repeated light-evoked synaptic stimuli caused weakening of visual responses in *Aqp4* KO mice. A similar fatigue phenomenon in response to repeated stimuli is observed in myasthenia gravis [39], where the autoimmune system alters the neurotransmitter refractory period in the neuromuscular junction. These findings support the idea that *Aqp4* deficiency causes a disorganization in the kinetics of neurotransmitters.

Extracellular potassium is known to be critical in defining the membrane potential of neurons [25]. Previous reports have shown that light-evoked increases in extracellular potassium in the OPL cause hyperactive ERG responses [40], while increased ERG amplitudes are also observed after injection of tetraethylammonium chloride, a blocker of potassium rectifiers [41]. We found that *Aqp4* KO mice show repression of *Kir2.1*, an inward potassium channel in the retina, and a previous report indicated increased extracellular potassium ion levels in the brain of *Aqp4* KO mice [42], suggesting that potassium ions may be also increased in KO retinas. Moreover, we showed that intraocular injection of KCL increased the ERG amplitude, similarly to *Aqp4* deletion in mice, supporting that hyperactive ERG responses observed in *Aqp4* KO mice would, at least in part, be induced by the increased extracellular potassium ion levels in the retina.

Insufficient KIR4.1 action, most likely by its redistribution, which is related to *Aqp4* deficiency, was reported to affect osmotic condition in Müller glial cells [43–45]. KIR4.1 is expressed in the membranes of Müller glial cells surrounding the retinal vessels, the end feet facing the vitreous cavity, and within the nerve fiber layer, but not at the synaptic site, which was reported previously [32, 46]. In addition, KIR4.1 levels are affected by vascular diseases, such as ischemia [24] and diabetes [34], and have a major role in regulating the blood-retina barrier [19]. In the current study, we found that the mRNA levels of *Kir2.1* are downregulated in *Aqp4* KO retinas and *Aqp4*-deficient Müller glial cells in vitro and that the protein is distributed around synapses in the mouse retina. While KIR 2.1 was previously reported to be present in Müller glial cells and, most likely, neurons of the inner nuclear layer in the retina [32], in contrast to the brain where it is expressed in neurons rather than glia [47], its localization to the synaptic area had not been clarified. Our present study showed that KIR 2.1 can be found in Müller glial cells at the synaptic area, but not in the synapses of neuronal cells in the retina, as shown by high-magnification images. Therefore, potassium homeostasis at the synaptic area is at least in part regulated by AQP4-KIR2.1 interaction, although we cannot exclude the possibility that other potassium channels may be involved in synaptic activity, in collaboration with AQP4.

Extracellular potassium ion levels affect electrogenic glutamate uptake [48]. We found that *Glast* is repressed in *Aqp4* KO retinas; this transporter in Müller glial cells uptakes the extracellular glutamate around photoreceptor cells and is required for glutamate recycling and preserving normal glutamate levels at the synaptic cleft between photoreceptors and bipolar cells [49]. Given that the mice showed hyperactive ERG responses, glutamate may have been accumulated in the synaptic cleft. Further, Müller glial cells not only prevent the diffusion of glutamate released by photoreceptor cells out of the synaptic cleft and glutamate uptake but also supply neurons with glutamine and neurotransmitter precursors, such as GABA [50, 51]. *Aqp4* KO mice exhibited lower levels of *Gs*, an enzyme that converts glutamate into glutamine, a source of GABA; visual hyperactivity observed in the KO mice may have involved the influence of GABA levels [52]. In addition, synaptic fatigue during repeated stimulation may be related to the insufficient clearance of glutamate at the synaptic area. Thus, *Aqp4* KO mice may have an imbalance in neurotransmitter metabolism. Regarding the a-wave derived from photoreceptor cells in scotopic ERG, the accumulated glutamate at the synaptic cleft during dark may have caused excessive depolarization of photoreceptor cells, thus resulting in an apparently greater amplitude at the time of the light stimulus.

Hyperactivity is observed at the time of synaptic remodeling related to local neurodegenerative changes [52]. While *Aqp4* KO mice show reduction in visual function at 40 weeks of age, as previously reported [26], the changes in the visual responses of the *Aqp4* KO mice at 12 and 16 weeks old were not the reduction but the hyperactivity and synaptic fatigue; these changes would have represented the early changes of the neural disorder. Neural hyperactivity is also observed at the early stage of Alzheimer’s disease, a neural disorder [53, 54]. Given that abnormal clearance of synaptic glutamate by Müller glial cells causes neurotoxicity [50, 55–58], and apoptotic markers were increased in *Aqp4* KO retinas, synaptic disorder and postsynaptic remodeling may have already occurred at least by 16 weeks of age, and thus *synaptophysin* expression was changed. The fact that neural hyperactivity precedes neurodegeneration is a common finding in Alzheimer’s disease and *Aqp4* deficiency. Thus, the current study may help understand the mechanism underlying Alzheimer’s disease [22].

Another similarity to Alzheimer’s disease involves mitochondria changes. Mitochondrial dysfunction in the brains of patients with Alzheimer’s disease correlates with a wide range of mitochondrial abnormalities. One hypothesis regarding such secondary abnormalities is that mitochondria aging is accelerated by neural hyperactivity, which further induces mitochondria exhaustion [59], given that synaptic transmission is regulated by mitochondria through modulating ATP production [60]. Moreover, dysregulation of mitochondria fission-fusion balance promotes neurodegeneration [61], and *Pgc1α* KO mice show hyperactivity [62]. In the retina of *Aqp4* KO mice, fission-fusion markers and *Pgc1α* were downregulated, which may have accelerated neurodegeneration in these mice. Therefore, a vicious cycle of hyperactivity and mitochondria disorganization might explain the pathogenesis of *Aqp4* KO mice and the age-related progression, leading to the induction of mitochondria-related apoptotic markers, like Bcl2 family molecules, in the retina of *Aqp4* KO mice.

In humans, hyperactivity in ERG responses is observed in central retinal vein occlusion [63], and the estimated mechanism involves ischemia-related increase in the local levels of inflammatory cytokines [64]. Given that AQP4 is important for water diffusion allowing bidirectional water transport across the plasma membrane [3, 12], the fine-tuning of diffusible factor concentrations, besides glutamate and potassium, at the neuron-glial site could be also involved in its role in preserving neuron-microenvironment homeostasis. Thus, multiple systems may be dysregulated in *Aqp4* KO retinas.

In summary, we demonstrated that neural hyperactivity and synaptic fatigue in *Aqp4* KO mice occur before neurodegeneration becomes evident. Moreover, we showed that *Aqp4* deficiency disturbs synaptic homeostasis and mitochondria metabolism most likely through dysregulation of potassium metabolism and possibly through glutamate kinetics. Our results suggest that the bidirectional water channel AQP4 is important for adjusting the levels of extracellular factors secreted upon neuronal firing and fine-tuning neurotransmission to regulate neural and visual activity.

## Electronic Supplementary Material

ESM 1 (DOCX 17 kb)

## References

[1] Agre P, Preston GM, Smith BL, Jung JS, Raina S, Moon C, Guggino WB, Nielsen S (1993). Aquaporin CHIP: the archetypal molecular water channel. Am J Phys.

[2] Brown D (2017). The discovery of water channels (aquaporins). Ann Nutr Metab.

[3] Yu YC, Sohma Y, Takimoto S, Miyauchi T, Yasui M (2013). Direct visualization and quantitative analysis of water diffusion in complex biological tissues using CARS microscopy. Sci Rep.

[4] Watanabe-Matsumoto S, Moriwaki Y, Okuda T, Ohara S, Yamanaka K, Abe Y, Yasui M, Misawa H (2018). Dissociation of blood-brain barrier disruption and disease manifestation in an aquaporin-4-deficient mouse model of amyotrophic lateral sclerosis. Neurosci Res.

[5] Ikeshima-Kataoka H, Abe Y, Abe T, Yasui M (2013). Immunological function of aquaporin-4 in stab-wounded mouse brain in concert with a pro-inflammatory cytokine inducer, osteopontin. Mol Cell Neurosci.

[6] Li XM, Wendu RL, Yao J, Ren Y, Zhao YX, Cao GF, Qin J, Yan B (2014). Abnormal glutamate metabolism in the retina of aquaporin 4 (AQP4) knockout mice upon light damage. Neurol Sci.

[7] Jo AO, Ryskamp DA, Phuong TT, Verkman AS, Yarishkin O, MacAulay N, Krizaj D (2015). TRPV4 and AQP4 channels synergistically regulate cell volume and calcium homeostasis in retinal Muller glia. J Neurosci.

[8] Pisani F, Cammalleri M, Dal Monte M, Locri F, Mola MG, Nicchia GP, Frigeri A, Bagnoli P, Svelto M (2018). Potential role of the methylation of VEGF gene promoter in response to hypoxia in oxygen-induced retinopathy: beneficial effect of the absence of AQP4. J Cell Mol Med.

[9] Ma T, Yang B, Gillespie A, Carlson EJ, Epstein CJ, Verkman AS (1997). Generation and phenotype of a transgenic knockout mouse lacking the mercurial-insensitive water channel aquaporin-4. J Clin Invest.

[10] Jung JS, Bhat RV, Preston GM, Guggino WB, Baraban JM, Agre P (1994). Molecular characterization of an aquaporin cDNA from brain: candidate osmoreceptor and regulator of water balance. Proc Natl Acad Sci U S A.

[11] Kozono D, Yasui M, King LS, Agre P (2002). Aquaporin water channels: atomic structure molecular dynamics meet clinical medicine. J Clin Invest.

[12] Nagelhus EA, Ottersen OP (2013). Physiological roles of aquaporin-4 in brain. Physiol Rev.

[13] Misu T, Fujihara K, Kakita A, Konno H, Nakamura M, Watanabe S, Takahashi T, Nakashima I, Takahashi H, Itoyama Y (2007). Loss of aquaporin 4 in lesions of neuromyelitis optica: distinction from multiple sclerosis. Brain J Neurol.

[14] Wingerchuk DM, Banwell B, Bennett JL, Cabre P, Carroll W, Chitnis T, de Seze J, Fujihara K, Greenberg B, Jacob A, Jarius S, Lana-Peixoto M, Levy M, Simon JH, Tenembaum S, Traboulsee AL, Waters P, Wellik KE, Weinshenker BG (2015). International consensus diagnostic criteria for neuromyelitis optica spectrum disorders. Neurology.

[15] Kezuka T, Usui Y, Yamakawa N, Matsunaga Y, Matsuda R, Masuda M, Utsumi H, Tanaka K, Goto H (2012). Relationship between NMO-antibody and anti-MOG antibody in optic neuritis. J Neuroophthalmol.

[16] Vujosevic S, Micera A, Bini S, Berton M, Esposito G, Midena E (2015). Aqueous humor biomarkers of Muller cell activation in diabetic eyes. Invest Ophthalmol Vis Sci.

[17] Cui B, Sun JH, Xiang FF, Liu L, Li WJ (2012). Aquaporin 4 knockdown exacerbates streptozotocin-induced diabetic retinopathy through aggravating inflammatory response. Exp Eye Res.

[18] Kumar B, Gupta SK, Nag TC, Srivastava S, Saxena R, Jha KA, Srinivasan BP (2014). Retinal neuroprotective effects of quercetin in streptozotocin-induced diabetic rats. Exp Eye Res.

[19] Zhang Y, Xu G, Ling Q, Da C (2011). Expression of aquaporin 4 and Kir4.1 in diabetic rat retina: treatment with minocycline. J Int Med Res.

[20] Jukkola P, Gu C (2015). Regulation of neurovascular coupling in autoimmunity to water and ion channels. Autoimmun Rev.

[21] Camassa LMA, Lunde LK, Hoddevik EH, Stensland M, Boldt HB, De Souza GA, Ottersen OP, Amiry-Moghaddam M (2015). Mechanisms underlying AQP4 accumulation in astrocyte endfeet. Glia.

[22] Lan YL, Zhao J, Ma T, Li S (2016). The potential roles of aquaporin 4 in Alzheimer’s disease. Mol Neurobiol.

[23] Haj-Yasein NN, Jensen V, Ostby I, Omholt SW, Voipio J, Kaila K, Ottersen OP, Hvalby O, Nagelhus EA (2012). Aquaporin-4 regulates extracellular space volume dynamics during high-frequency synaptic stimulation: a gene deletion study in mouse hippocampus. Glia.

[24] Iandiev I, Tenckhoff S, Pannicke T, Biedermann B, Hollborn M, Wiedemann P, Reichenbach A, Bringmann A (2006). Differential regulation of Kir4.1 and Kir2.1 expression in the ischemic rat retina. Neurosci Lett.

[25] Vecino E, Rodriguez FD, Ruzafa N, Pereiro X, Sharma SC (2016). Glia-neuron interactions in the mammalian retina. Prog Retin Eye Res.

[26] Li J, Patil RV, Verkman AS (2002). Mildly abnormal retinal function in transgenic mice without Muller cell aquaporin-4 water channels. Invest Ophthalmol Vis Sci.

[27] Kato J, Takai Y, Hayashi MK, Kato Y, Tanaka M, Sohma Y, Abe Y, Yasui M (2014). Expression and localization of aquaporin-4 in sensory ganglia. Biochem Biophys Res Commun.

[28] Ramadhanti J, Huang P, Kusano-Arai O, Iwanari H, Sakihama T, Misu T, Fujihara K, Hamakubo T, Yasui M, Abe Y (2013). A novel monoclonal antibody against the C-terminal region of aquaporin-4. Monoclon Antib Immunodiagn Immunother.

[29] Tanimoto N, Sothilingam V, Kondo M, Biel M, Humphries P, Seeliger MW (2015). Electroretinographic assessment of rod- and cone-mediated bipolar cell pathways using flicker stimuli in mice. Sci Rep.

[30] Wang M, Ma W, Zhao L, Fariss RN, Wong WT (2011). Adaptive Muller cell responses to microglial activation mediate neuroprotection and coordinate inflammation in the retina. J Neuroinflammation.

[31] Fontaine V, Mohand-Said S, Hanoteau N, Fuchs C, Pfizenmaier K, Eisel U (2002) Neurodegenerative and neuroprotective effects of tumor necrosis factor (TNF) in retinal ischemia: opposite roles of TNF receptor 1 and TNF receptor 2 J Neurosci 22(7):RC216. doi:20026253 10.1523/JNEUROSCI.22-07-j0001.2002 PMC6758303 11917000

[32] Kofuji P, Biedermann B, Siddharthan V, Raap M, Iandiev I, Milenkovic I, Thomzig A, Veh RW, Bringmann A, Reichenbach A (2002). Kir potassium channel subunit expression in retinal glial cells: implications for spatial potassium buffering. Glia.

[33] Raap M, Biedermann B, Braun P, Milenkovic I, Skatchkov SN, Bringmann A, Reichenbach A (2002). Diversity of Kir channel subunit mRNA expressed by retinal glial cells of the Guinea-pig. Neuroreport.

[34] Bringmann A, Pannicke T, Grosche J, Francke M, Wiedemann P, Skatchkov SN, Osborne NN, Reichenbach A (2006). Muller cells in the healthy and diseased retina. Prog Retin Eye Res.

[35] Aslanidis A, Karlstetter M, Walczak Y, Jagle H, Langmann T (2014). RETINA-specific expression of Kcnv2 is controlled by cone-rod homeobox (Crx) and neural retina leucine zipper (Nrl). Adv Exp Med Biol.

[36] Skatchkov SN, Eaton MJ, Shuba YM, Kucheryavykh YV, Derst C, Veh RW, Wurm A, Iandiev I, Pannicke T, Bringmann A, Reichenbach A (2006). Tandem-pore domain potassium channels are functionally expressed in retinal (Muller) glial cells. Glia.

[37] Hurley JB, Lindsay KJ, Du J (2015). Glucose, lactate, and shuttling of metabolites in vertebrate retinas. J Neurosci Res.

[38] Country MW (2017). Retinal metabolism: a comparative look at energetics in the retina. Brain Res.

[39] Pasnoor M, Dimachkie MM, Farmakidis C, Barohn RJ (2018). Diagnosis of myasthenia gravis. Neurol Clin.

[40] Karwoski CJ, Newman EA, Shimazaki H, Proenza LM (1985). Light-evoked increases in extracellular K+ in the plexiform layers of amphibian retinas. J Gen Physiol.

[41] Lei B, Perlman I (1999). The contributions of voltage- and time-dependent potassium conductances to the electroretinogram in rabbits. Vis Neurosci.

[42] Haj-Yasein NN, Bugge CE, Jensen V, Ostby I, Ottersen OP, Hvalby O, Nagelhus EA (2015). Deletion of aquaporin-4 increases extracellular K(+) concentration during synaptic stimulation in mouse hippocampus. Brain Struct Funct.

[43] Rehak M, Hollborn M, Iandiev I, Pannicke T, Karl A, Wurm A, Kohen L, Reichenbach A, Wiedemann P, Bringmann A (2009). Retinal gene expression and Muller cell responses after branch retinal vein occlusion in the rat. Invest Ophthalmol Vis Sci.

[44] Iandiev I, Wurm A, Hollborn M, Wiedemann P, Grimm C, Reme CE, Reichenbach A, Pannicke T, Bringmann A (2008). Muller cell response to blue light injury of the rat retina. Invest Ophthalmol Vis Sci.

[45] Ruiz-Ederra J, Zhang H, Verkman AS (2007). Evidence against functional interaction between aquaporin-4 water channels and Kir4.1 potassium channels in retinal Muller cells. J Biol Chem.

[46] Ji M, Miao Y, Dong LD, Chen J, Mo XF, Jiang SX, Sun XH, Yang XL, Wang Z (2012). Group I mGluR-mediated inhibition of Kir channels contributes to retinal Muller cell gliosis in a rat chronic ocular hypertension model. J Neurosci.

[47] Pruss H, Derst C, Lommel R, Veh RW (2005). Differential distribution of individual subunits of strongly inwardly rectifying potassium channels (Kir2 family) in rat brain. Brain Res Mol Brain Res.

[48] Pannicke T, Faude F, Reichenbach A, Reichelt W (2000). A function of delayed rectifier potassium channels in glial cells: maintenance of an auxiliary membrane potential under pathological conditions. Brain Res.

[49] Harada T, Harada C, Watanabe M, Inoue Y, Sakagawa T, Nakayama N, Sasaki S, Okuyama S, Watase K, Wada K, Tanaka K (1998). Functions of the two glutamate transporters GLAST and GLT-1 in the retina. Proc Natl Acad Sci U S A.

[50] Reichenbach A, Bringmann A (2013). New functions of Muller cells. Glia.

[51] Bringmann A, Pannicke T, Biedermann B, Francke M, Iandiev I, Grosche J, Wiedemann P, Albrecht J, Reichenbach A (2009). Role of retinal glial cells in neurotransmitter uptake and metabolism. Neurochem Int.

[52] Soto F, Kerschensteiner D (2015). Synaptic remodeling of neuronal circuits in early retinal degeneration. Front Cell Neurosci.

[53] Busche MA, Chen X, Henning HA, Reichwald J, Staufenbiel M, Sakmann B, Konnerth A (2012). Critical role of soluble amyloid-beta for early hippocampal hyperactivity in a mouse model of Alzheimer’s disease. Proc Natl Acad Sci U S A.

[54] Busche MA, Eichhoff G, Adelsberger H, Abramowski D, Wiederhold KH, Haass C, Staufenbiel M, Konnerth A, Garaschuk O (2008). Clusters of hyperactive neurons near amyloid plaques in a mouse model of Alzheimer’s disease. Science.

[55] Reichenbach A, Derouiche A, Kirchhoff F (2010). Morphology and dynamics of perisynaptic glia. Brain Res Rev.

[56] Bringmann A, Uckermann O, Pannicke T, Iandiev I, Reichenbach A, Wiedemann P (2005). Neuronal versus glial cell swelling in the ischaemic retina. Acta Ophthalmol Scand.

[57] Izumi Y, Kirby CO, Benz AM, Olney JW, Zorumski CF (1999). Muller cell swelling, glutamate uptake, and excitotoxic neurodegeneration in the isolated rat retina. Glia.

[58] Izumi Y, Shimamoto K, Benz AM, Hammerman SB, Olney JW, Zorumski CF (2002). Glutamate transporters and retinal excitotoxicity. Glia.

[59] Jeong S (2017). Molecular and cellular basis of neurodegeneration in Alzheimer’s disease. Mol Cell.

[60] Gazit N, Vertkin I, Shapira I, Helm M, Slomowitz E, Sheiba M, Mor Y, Rizzoli S, Slutsky I (2016). IGF-1 receptor differentially regulates spontaneous and evoked transmission via mitochondria at hippocampal synapses. Neuron.

[61] Faits MC, Zhang C, Soto F, Kerschensteiner D (2016) Dendritic mitochondria reach stable positions during circuit development. eLife 5:e11583. doi:10.7554/eLife.11583 10.7554/eLife.11583 PMC4749546 26742087

[62] Lin J, Wu PH, Tarr PT, Lindenberg KS, St-Pierre J, Zhang CY, Mootha VK, Jager S, Vianna CR, Reznick RM, Cui L, Manieri M, Donovan MX, Wu Z, Cooper MP, Fan MC, Rohas LM, Zavacki AM, Cinti S, Shulman GI, Lowell BB, Krainc D, Spiegelman BM (2004). Defects in adaptive energy metabolism with CNS-linked hyperactivity in PGC-1alpha null mice. Cell.

[63] Gouras P, MacKay CJ (1992). Supernormal cone electroretinograms in central retinal vein occlusion. Invest Ophthalmol Vis Sci.

[64] Miyata R, Kondo M, Kato K, Sugimoto M, Matsubara H, Ikesugi K, Ueno S, Yasuda S, Terasaki H (2018). Supernormal flicker ERGs in eyes with central retinal vein occlusion: clinical characteristics, prognosis, and effects of anti-VEGF agent. Invest Ophthalmol Vis Sci.

