# Supplementary material for: Aquaporin 4 Suppresses Neural Hyperactivity and Synaptic Fatigue and Fine-Tunes Neurotransmission to Regulate Visual Function in the Mouse Retina
Source: Mol Neurobiol. 2019 Jun 12;56(12):8124–35. doi: 10.1007/s12035-019-01661-2 (PMC6834759; doi:10.1007/s12035-019-01661-2)
Supplement: Supplementary file 1 — (DOCX 17 kb) [file 12035_2019_1661_MOESM1_ESM.docx]

| **Supplementary Table 1. Measurements of ERG responses.** | | | | | |
| --- | --- | --- | --- | --- | --- |
| **Scotopic ERG (12 weeks old)** | | | | | |
| a-wave Amplitude (mV) | | | | | |
| Intensity | -2.1 | -0.6 | 0.4 | 1.9 | 2.9 |
| WT | 20.1 ± 11.3 | 257.0 ± 39.3 | 427.2 ± 58.0 | 581.4 ± 58.4 | 677.5 ± 71.7 |
| KO | 28.3 ± 5.3 | 294.0 ± 40.3 | 499.5 ± 49.5 | 656.6 ± 84.0 | 770.5 ± 106.1 |
| p value | 0.199 | 0.179 | 0.068 | 0.143 | 0.148 |
| a-wave Implicit time (ms) | | | | | |
| Intensity | -2.1 | -0.6 | 0.4 | 1.9 | 2.9 |
| WT | 22.1 ± 1.7 | 18.4 ± 0.9 | 10.6 ± 0.4 | 6.4 ± 0.2 | 4.7 ± 0.1 |
| KO | 20.6 ± 1.1 | 17.8 ± 0.4 | 10.5 ± 0.1 | 6.2 ± 0.1 | 4.6 ± 0.1 |
| p value | 0.140 | 0.245 | 0.764 | 0.355 | 0.193 |
|  |  |  |  |  |  |
| b-wave Amplitude (mV) | | | | | |
| Intensity | -2.1 | -0.6 | 0.4 | 1.9 | 2.9 |
| WT | 349.9 ± 99.8 | 453.1 ± 117.5 | 670.0 ± 130.3 | 865.0 ± 121.9 | 967.1 ± 108.6 |
| KO | 468.0 ± 62.4 | 592.5 ± 86.8 | 854.1 ± 94.6 | 1046.7 ± 116.2 | 1221.2 ± 167.1 |
| p value | 0.061 | 0.068 | 0.036* | 0.042* | 0.025* |
| b-wave Implicit time (ms) | | | | | |
| Intensity | -2.1 | -0.6 | 0.4 | 1.9 | 2.9 |
| WT | 69.8 ± 12.9 | 56.4 ± 14.2 | 69.9 ± 8.5 | 74.7 ± 4.9 | 80.5 ± 6.3 |
| KO | 75.5 ± 7.8 | 60.8 ± 14.1 | 65.1 ± 9.3 | 68.6 ± 6.0 | 70.9 ± 7.6 |
| p value | 0.436 | 0.631 | 0.417 | 0.116 | 0.064 |
|  |  |  |  |  |  |
| **Scotopic ERG (16 weeks old)** | | | | | |
| a-wave Amplitude (mV) | | | | | |
| Intensity | -2.1 | -0.6 | 0.4 | 1.9 | 2.9 |
| WT | 24.1 ± 4.8 | 240.8 ± 26.8 | 392.9 ± 54.5 | 526.3 ± 55.7 | 570.8 ± 53.3 |
| KO | 32.6 ± 3.2 | 296.8 ± 41.2 | 512.4 ± 36.4 | 659.8 ± 44.3 | 736.0 ± 51.6 |
| p value | 0.028* | 0.044* | 0.013* | 0.009** | 0.003** |
| a-wave Implicit time (ms) | | | | | |
| Intensity | -2.1 | -0.6 | 0.4 | 1.9 | 2.9 |
| WT | 21.9 ± 3.2 | 19.0 ± 1.9 | 10.4 ± 0.5 | 6.4 ± 0.4 | 4.7 ± 0.3 |
| KO | 22.1 ± 1.0 | 18.1 ± 0.7 | 10.6 ± 0.3 | 6.3 ± 0.2 | 4.6 ± 0.1 |
| p value | 0.897 | 0.437 | 0.527 | 0.800 | 0.580 |
|  |  |  |  |  |  |
|  |  |  |  |  |  |
| b-wave Amplitude (mV) | | | | | |
| Intensity | -2.1 | -0.6 | 0.4 | 1.9 | 2.9 |
| WT | 307.4 ± 82.5 | 432.4 ± 79.1 | 590.5 ± 121.2 | 768.3 ± 134.4 | 801.5 ± 104.6 |
| KO | 464.9 ± 30.0 | 597.5 ± 38.3 | 840.7 ± 66.3 | 1064.5 ± 58.1 | 1123.1 ± 90.1 |
| p value | 0.026* | 0.018* | 0.017* | 0.015* | 0.003** |
| b-wave Implicit time (ms) | | | | | |
| Intensity | -2.1 | -0.6 | 0.4 | 1.9 | 2.9 |
| WT | 63.9 ± 6.8 | 49.6 ± 9.3 | 48.5 ± 12.0 | 56.7 ± 17.9 | 58.5 ± 20.1 |
| KO | 72.6 ± 3.3 | 57.5 ± 1.8 | 64.6 ± 4.7 | 69.3 ± 6.0 | 64.3 ± 2.1 |
| p value | 0.078 | 0.188 | 0.069 | 0.256 | 0.608 |
|  |  |  |  |  |  |
| **Photopic ERG (16 weeks old)** | | | | | |
| b-wave Amplitude (μV) | | |  |  |  |
| Intensity | 0.4 | 1.4 |  |  |  |
| WT | 148.9 ± 22.1 | 178.5 ± 8.4 |  |  |  |
| KO | 221.2 ± 36.5 | 280.1 ± 9.0 |  |  |  |
| p value | 0.069 | 0.045* |  |  |  |
| b-wave Implicit time (ms) | | |  |  |  |
| Intensity | 0.4 | 1.4 |  |  |  |
| WT | 37.8 ± 2.0 | 36.2 ± 2.1 |  |  |  |
| KO | 36.4 ± 0.4 | 35.1 ± 0.4 |  |  |  |
| p value | 0.268 | 0.373 |  |  |  |
| Data are shown in mean ± standard deviation. Intensity, Stimulus intensity (log cd sec/ m2). *p<0.05, **p<0.01. | | | | | |
